# Emulation of a Target Trial of Antihypertensive Medications on Weight Change

**DOI:** 10.1007/s11606-025-09787-x

**Published:** 2025-09-29

**Authors:** Pi-I D. Lin, Sheryl L. Rifas-Shiman, Joshua Petimar, Han Yu, Matthew F. Daley, David M. Janicke, William J. Heerman, L. Charles Bailey, Carlos Maeztu, Kristina H. Lewis, Jessica G. Young, Jason P. Block

**Affiliations:** 1 Division of Chronic Disease Research Across the Lifecourse, Department of Population Medicine, Harvard Medical School and Harvard Pilgrim Health Care Institute, Boston, MA USA; 2 Department of Epidemiology, Harvard TH Chan School of Public Health, Boston, MA USA; 3 Institute for Health Research, Kaiser Permanente Colorado, Aurora, CO USA; 4 Department of Clinical and Health Psychology, University of Florida, Gainesville, FL USA; 5 Department of Pediatrics, Vanderbilt University Medical Center, Nashville, TN USA; 6 Applied Clinical Research Center, Children’s Hospital of Philadelphia, Philadelphia, PA USA; 7 Department of Health Outcomes and Biomedical Informatics, University of Florida, Gainesville, FL USA; 8 Department of Epidemiology and Prevention, Wake Forest University School of Medicine, Winston-Salem, NC USA

**Keywords:** Weight, Hypertension, Epidemiology, Target trial, Electronic health records

## Abstract

**Background:**

Weight gain after starting antihypertensive medications is a frequent concern for patients, but there is limited data on expected weight change after initiation of these medications. A comparative effectiveness trial to evaluate this outcome would not be feasible.

**Objective:**

To estimate and compare average weight change under initiating and adhering to commonly prescribed, first-line antihypertensive medications as monotherapy by emulating a target trial.

**Design:**

Retrospective observational cohort study over 24 months of follow-up using electronic health records (EHR).

**Participants:**

141,260 patients prescribed one of seven antihypertensives between 2010 and 2019 across 8 US health systems.

**Main Outcome and Measures:**

We examined mean weight change associated with initiation of and adherence to amlodipine, atenolol, hydrochlorothiazide, losartan, metoprolol, or propranolol, relative to lisinopril, at 6, 12, and 24 months after initiation. To adjust for baseline confounding and informative outcome measurement, we used inverse probability weighting with repeated outcome marginal structural models.

**Key Results:**

After baseline and time-varying covariate adjustment, initiation of and adherence to lisinopril were associated with mean weight loss at 6 months (− 0.69 kg, 95% CI − 0.92, − 0.47), 12 months (− 0.58 kg, 95% CI − 1.05, − 0.30), and 24 months (− 1.121 kg, 95% CI − 2.013, − 0.46). Compared to lisinopril, the estimated 6-month weight change was higher for patients prescribed hydrochlorothiazide (0.68 kg, 95% CI 0.31, 1.04), losartan (0.54 kg, 95% CI 0.17, 0.93), metoprolol (1.38 kg, 95% CI 0.95, 1.76), and propranolol (1.03 kg, 95% CI 0.346, 1.62). At 12 months, metoprolol (1.74 kg, 95% CI 1.03, 2.41) and propranolol (1.72 kg, 95% CI 0.06, 3.235) continued to show higher weight change compared to lisinopril.

**Conclusion:**

We observed small differences in weight change across antihypertensive medications, with lisinopril leading to weight loss and metoprolol and propranolol to modest weight gain. Clinicians should consider potential weight gain when selecting antihypertensive medications.

**Supplementary Information:**

The online version contains supplementary material available at 10.1007/s11606-025-09787-x.

## INTRODUCTION

Hypertension is a leading cause of global morbidity and mortality.^1^ Effective treatment with antihypertensives can reduce these outcomes.^2^ In the USA, antihypertensive use increased by 3% from 2017 to 2021^3^, likely due to updated guidelines that lowered hypertension thresholds.^4^ While antihypertensives are effective for blood pressure control and other reducing symptoms for conditions like migraines^5,6^, they can be associated with adverse events, including weight gain.^7,8^

Antihypertensive subclasses affect body weight differently^8–10^: beta-blockers (e.g., atenolol, metoprolol, propranolol) are linked with weight gain; calcium channel blockers are typically weight-neutral; and angiotensin II receptor blockers (ARBs), angiotensin-converting enzyme (ACE) inhibitors, and diuretics may promote weight loss. However, existing evidence^11–14^, mainly from small (*n* < 100), older randomized controlled trials (RCTs), is sparse and has limited generalizability.

Conducting a sufficiently large RCT to assess weight change associated with various antihypertensive medications is not feasible. Instead, evidence can be generated by replicating target trials using healthcare data.^15–18^ In this study, we aimed to emulate a target trial using electronic health record (EHR) data to compare the effects of common antihypertensive medications on weight change over 6, 12, and 24 months. We hypothesized that initiating and adhering to ACE inhibitors, ARBs, and thiazide diuretics would be associated with weight loss while beta-blockers would be associated with weight gain.

## METHODS

### Target Trial Overview

We implemented a target trial emulation using EHR data to clarify the causal question.^15,16,19^ Target trial emulation is a methodological framework that aligns observational data with RCT principles to enhance causal inference. By specifying a hypothetical target trial—including eligibility criteria, treatment strategies, follow-up, and outcomes—we aimed to identify and better account for biases like baseline and time-varying confounding and informative outcome measurements.^19^

In this target trial (Table 1), we would enroll adults aged 20–80 years planning to start one of seven common antihypertensive monotherapies, excluding those with prior antihypertensive use or conditions or medication use that could substantially influence weight (e.g., cancer, pregnancy, bariatric surgery, heart failure, use of anti-obesity medications, steroids, or stimulants). The seven antihypertensives would be selected based on their high prescription frequency^20^, ensuring that they were reflective of clinical practice and to allow for robust comparisons with sufficient sample size. Participants would have a baseline weight measure and be randomly assigned to one of the treatments. The primary outcome would be weight change at 6, 12, and 24 months post randomization. The effects of interest would be (1) per-protocol (PP) effects, reflecting the impact of continuous adherence, and (2) initiation-only (IO) effects, reflecting the impact of starting treatment regardless of adherence (see Table S1 and our previous publication^21^ for more details).

**Table 1:** Specifications of the Target Trial and Emulation with Observational Electronic Health Record Data

Protocol component	Target trial specification	Emulation
Eligibility criteria	• Age 20 to ≤ 80 years • No prior antihypertensive use • Available weight measurement at baseline • Indicated for monotherapy of one of the considered medications • Exclusion of conditions that could inference weight: ▹No cancer diagnosis (other than non-melanoma skin cancer) 1 year prior to initiation and 1 month after initiation ▹No pregnancy 1 year prior to initiation ▹No bariatric surgery 3 years prior to initiation and 1 month after initiation ▹No heart failure 1 year prior to initiation and 1 month after initiation ▹No use of anti-obesity medications, steroids, or stimulants 1 month prior to initiation and 1 month after initiation	• Age 20 to ≤ 80 years • ≥ 1 encounter at least 6 months prior to first prescription • Weight measured within a 3-month period prior to initiation • Initiated only one of the considered medications • No cancer diagnosis (other than non-melanoma skin cancer) 1 year prior to initiation and 1 month after initiation • No pregnancy 1 year prior to initiation • No bariatric surgery 3 years prior to initiation and 1 month after initiation • No heart failure 1 year prior to initiation and 1 month after initiation • No use of anti-obesity medications, steroids, or stimulants 1 month prior to initiation and 1 month after initiation
Baseline	• Randomization upon meeting all eligibility criteria	• Baseline is the date of treatment initiation when all eligibility criteria are met
Treatment strategies	• **IO**: Initiate only 1 of the following medications: (1) lisinopril, (2) amlodipine, (3) atenolol, (4) hydrochlorothiazide, (5) losartan, (6) metoprolol, or (7) propranolol • **PP**: Initiate and adhere to the assigned medication on a daily basis, allowing for a 1-month “grace period” (i.e., allowing the patient to go 1 month without taking the medication, but no longer)	• Date of medication initiation was the date of first prescription • We estimated the amount of time a patient had medication using information on number of pills, days supply, and number of refills from the prescription. Patients were considered adherent during the time when they had medication on hand based on these calculations; the month following the end of their supply of medicine was the grace period
Treatment assignment	• Randomly assigned to a treatment strategy at baseline	• Treatment not assigned randomly (requires confounding adjustment)
Outcome	• Weight change compared to baseline weight after 6, 12, 24 months	• Same as target trial
Follow-up period	• Starts at baseline and ends at the end of available data, death, or 2 years after baseline	• Same as target trial
Analysis plan	• **IO**: Calculate change in weight from baseline to each timepoint • **PP**: censor patients when they deviate from their assigned treatment strategy and apply inverse probability weights to adjust for factors associated with adherence/treatment discontinuation	• **IO**: Adjust for baseline covariates and apply inverse probability weights to adjust for informative outcome measurement. Predict weight change had each patient initiated and adhered to each medication of interest at each time point *t* • **PP**: same as IO but censor patients when they deviate from their treatment strategy and modify inverse probability weights to additionally adjust for factors associated with adherence/treatment discontinuation
Contrast of interest	• Mean weight change for each antihypertensive compared with that of lisinopril	• Same as target trial

Abbreviations: *IO *Inition-Only, *PP *Per Protocol

### Study Population

For the trial emulation (Table 1), we used EHR data from eight PCORnet institutions^20,22,23^ (see Table S2 for the full list and details on the data query in our Github Repository: https://github.com/PCORnet-DRN-OC/Query-Details/tree/master/MedWeight%20Project) including patients starting one of seven common antihypertensive between July 2010 and December 2019. For analysis, we aggregated demographic, prescriptions, and clinical data into 30-day person-months intervals relative to the initial prescription date.^21^

We extracted data on 2,355,075 patients prescribed antihypertensives and excluded patients who were prescribed multiple antihypertensives at initiation, prescribed medication subclasses not included in this study, or did not have a healthcare visit 6 months before starting the medication to ensure that we could best identify patients newly initiating common antihypertensives. Further exclusions included patients aged < 20 or > 80 years or those with conditions or medication prescriptions associated with rapid weight change. The higher age exclusion was intended to avoid circumstances where patients were experiencing sarcopenia and resulting weight change associated with aging. To facilitate analyses, we excluded patients lacking baseline weight data within 3 months before initiation, without race and ethnicity or sex information, and without documented blood pressure within 6 months before initiation. The final sample consisted of 141,260 patients. Exclusion details are provided in Figure S1.

The study was approved by the Institutional Review Board of Harvard Pilgrim Health Care.

### Treatment Strategies

We categorized patients based on their initiation of one of seven medications: lisinopril, amlodipine, atenolol, hydrochlorothiazide, losartan, metoprolol, or propranolol. Incident use was defined as the first prescription for these medications. A manual chart review of 36 records confirmed 80.5% accuracy in identifying new users.

Adherence was defined as consistent availability of medication for use that was consistent with the study protocol, measured using information from prescriptions over time, with allowance for a 1-month grace period at the end of the period when patients should have had available medication.^24^ We used information from prescription data (refills, pill quantity, days supply) for this measure. There was sufficient data for approximately 50% of all prescriptions and 48% at initiation. When available, we imputed missing data from other prescriptions for the same medication, prioritizing those from the closest date available. For prescriptions missing initiation data, we assumed a minimum prescription duration of one month.

### Outcome

We cleaned^25^ the EHR weight data using the R package *growthcleanr*^26,27^ to remove errors, duplicates, and inconsistencies. Baseline weight was the measurement closest to, but not after, medication initiation, within 3 months. For patients with multiple weight measurements in a month, we averaged the measurements. Monthly weight change was calculated by subtracting baseline weight from each month’s weight. The primary outcome was weight change at 6 months, with 12 and 24 months as secondary outcomes; however, we used all available monthly data to enhance precision. We also assessed weight gain of ≥ 5% or more from baseline.

### Covariates

We adjusted for baseline and time-varying covariates that were potentially associated with outcomes, medication initiation, and adherence. Baseline covariates included age, sex, race, ethnicity, year of medication initiation, Medicaid use, study site, underlying health conditions (identified by ICD codes), baseline blood pressure, recent weight change (≥ 0.5 kg gain, loss, or no change in the 6 months before baseline), smoking status, and medications affecting weight. Time-varying covariates included new prescriptions, healthcare utilization, cancer diagnoses, pregnancy, and bariatric surgery. For the full list of covariates, see Table S3.

### Statistical Analysis

We estimated both IO and PP effects over time using a particular application of inverse probability weighting (IPW) of marginal structural models. This approach was tailored to (1) estimate the effects of treatment initiation and adherence strategies over time, as explicitly defined in a trial protocol, and (2) account for measured baseline and time-varying confounding inherent in observational data.^28,29^ Primary analyses were based on PP effects which, compared to IO effects, mitigate capturing effects of differential adherence across medications. We utilized longitudinal data on eligible patients from treatment initiation until the end of a 24-month follow-up period or death (ascertained from the EHR, *n* = 1237), whichever occurred first. For IO analyses, we calculated IPWs^21,30,31^ to adjust for informative outcome measurement, incorporating baseline and time-varying covariates predicting outcome missingness in each month. To mitigate the influence of extreme weights, IPWs were truncated at the 99th percentile. We applied these IPWs to a repeated outcomes model, regressing weight change on treatment (antihypertensive medication), time (restricted cubic spline with 4 knots), and baseline covariates. Interaction terms for treatment by month were also included. For each medication at each selected follow-up month, model coefficients were used to predict weight change in that month under initiation of that treatment strategy, averaging predictions to obtain population-level estimates. Comparative effects were evaluated relative to lisinopril, the most commonly prescribed medication.^20^

For the PP analysis, we censored patients during follow-up if they became nonadherent (using a 1-month grace period). We calculated IPWs to account for both informative outcome measurement and selection bias due to censoring using baseline and time-varying covariates.^21^ We fit a repeated outcomes model to the censored data and estimated 95% confidence intervals using 1000 bootstrapped samples.

As secondary analyses, we stratified PP analyses by baseline obesity (BMI ≥ 30 kg/m^2^), sex (male vs. female), race (White vs. Non-White), and a proxy for menopausal status (women < 55 years vs. ≥ 55 years at baseline), and conducted a sensitivity analysis on patients with documented hypertension at baseline (presence of ICD diagnosis code) or elevated blood pressure (systolic ≥ 140 mmHg or diastolic ≥ 90 mmHg) within 6 months before treatment (*n* = 110,721). Additionally, we estimated the relative risk (RR) of gaining ≥ 5% of baseline weight compared to lisinopril.

All statistical modeling was performed using SAS Enterprise Guide, version 8.3, adhering to the Strengthening the Reporting of Observational Studies in Epidemiology (STROBE) guidelines.^32^

## RESULTS

**Figure 1:**
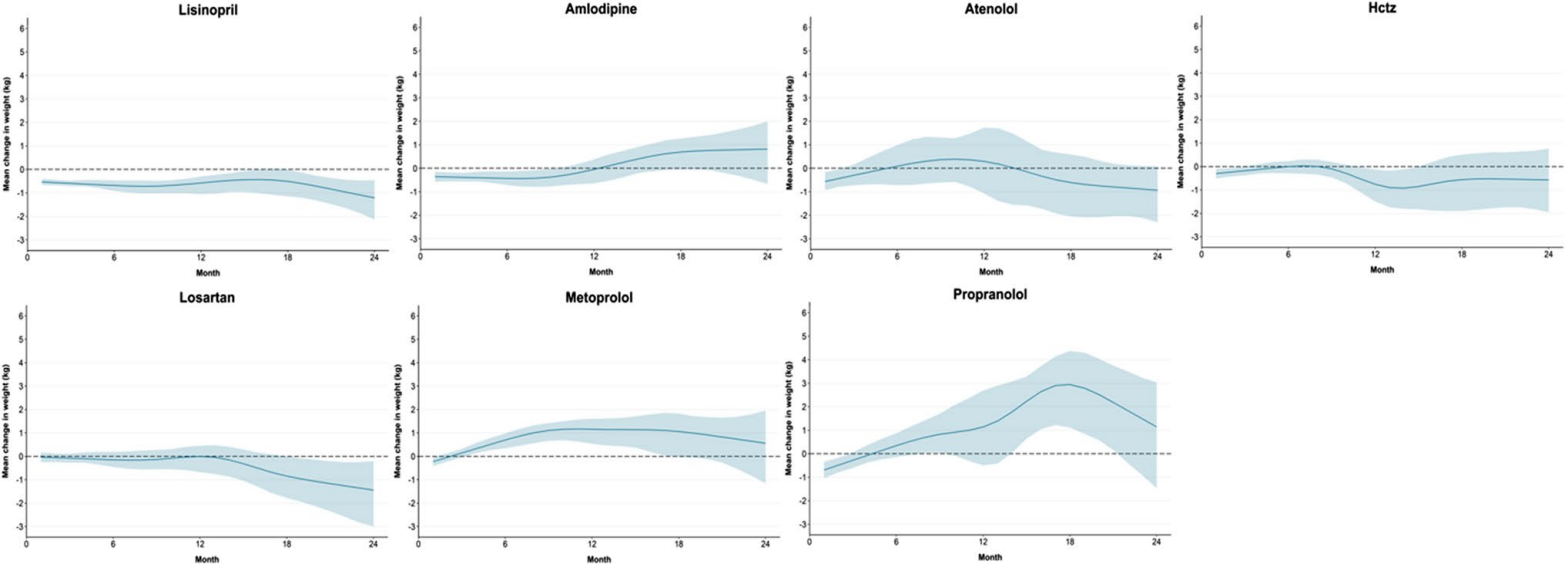
Per-protocol effect: estimated mean weight change (kg) and 95% confidence intervals associated with initiating and adhering to common antihypertensive medication over 24 months. The figure shows adjusted population-level average weight change estimates (dark blue line) and 95% confidence intervals from 1000 bootstrapped samples (light blue bands) for adherence to each of the 7 antihypertensive medications over 24 months from initiation. The null (0 kg mean weight change) is depicted with a dashed horizontal line. Abbreviation: Hctz, hydrochlorothiazide.

The study included 141,260 adults (56% female, 72% White, 20% Black) with a mean (SD) age of 53.3 (14.0) years and BMI of 30.6 (7.3) kg/m^2^ at baseline (Table 2). Medication adherence after initiation declined substantially over time (Table S4). Based on the PP analyses, we estimated that adherence to lisinopril and amlodipine was associated with a small absolute weight loss at 6 months (lisinopril − 0.69 kg, 95% CI: − 0.92, − 0.47; amlodipine − 0.43, 95% CI: − 0.72, − 0.14), and adherence to metoprolol with weight gain (0.70, 95% CI: 0.34, 0.98). We did not find evidence that other antihypertensives were associated with absolute weight change at 6 months (Fig. 1,Table 3). Compared to lisinopril, the most commonly prescribed antihypertensive, we estimated that initiation of and adherence to the following medications were associated with relative mean higher weight change at 6 months: hydrochlorothiazide (mean difference = 0.68 kg, 95% CI: 0.31, 1.04), losartan (difference = 0.54 kg, 95% CI: 0.17, 0.93), metoprolol (difference = 1.38 kg, 95% CI: 0.95, 1.76), and propranolol (difference = 1.03 kg, 95% CI: 0.46, 1.62). This higher weight change was persistent at 12 months for patients taking propranolol (difference = 1.72 kg, 95% CI: 0.06, 3.35) and metoprolol (difference = 1.74 kg, 95% CI: 1.03, 2.41) (Table 3). At 24 months, amlodipine (difference = 2.03 kg, 95% CI: 0.50, 3.57) would be associated with higher weight change compared to lisinopril. Across all outcome time periods, no weight change difference was evident for atenolol compared to lisinopril. However, the RR of gaining ≥ 5% baseline weight was significantly higher for atenolol compared to lisinopril at 6 months (RR = 1.68, 95% CI: 1.13, 2.67). Similarly, the RR for metoprolol was significantly elevated at 6 months (RR = 1.67, 95% CI: 1.33, 2.19) and 12 months (RR = 1.55, 95% CI: 1.18, 2.25).

**Table 2:** Baseline Participant Characteristics

Characteristic	Overall	Amlodipine	Atenolol	Hctz	Lisinopril	Losartan	Metoprolol	Propranolol
	*n* = 141,260	18,893 (13)	6375 (5)	17,326 (12)	51,502 (36)	9695 (7)	21,854 (15)	15,615 (11)
**Age in years, mean (SD)**	53.3 (14.0)	55.1 (13.8)	53.4 (15.1)	52.7 (13.2)	54.1 (12.6)	57.7 (12.3)	56.1 (14.4)	42.0 (14.4)
**Sex, *N* (%)**								
Male	62,578 (44)	8972 (47)	2250 (35)	5376 (31)	26,988 (52)	4484 (46)	9804 (45)	4704 (30)
Female	78,682 (56)	9921 (53)	4125 (65)	11,950 (69)	24,514 (48)	5211 (54)	12,050 (55)	10,911 (70)
**Race, *N* (%)**								
Asian American or Pacific Islander	3314 (2)	372 (2)	157 (2)	295 (2)	1499 (3)	267 (3)	333 (2)	391 (3)
Black or African American	28,356 (20)	6788 (36)	690 (11)	5574 (32)	9057 (18)	1934 (20)	2714 (12)	1599 (10)
White	102,242 (72)	10,838 (57)	5203 (82)	10,850 (63)	37,777 (73)	6986 (72)	17,776 (81)	12,812 (82)
Other or > 1 race	7348 (5)	895 (5)	325 (5)	607 (4)	3169 (6)	508 (5)	1031 (5)	813 (5)
**Ethnicity, *N* (%)**								
Hispanic	9985 (7)	1193 (6)	452 (7)	933 (5)	4079 (8)	859 (9)	1426 (7)	14,572 (93)
Not Hispanic	131,275 (93)	17,700 (94)	5923 (93)	16,393 (95)	47,423 (92)	8836 (91)	20,428 (93)	1043 (7)
**Weight in kg, mean (SD)**	88.47 (23.3)	88.52 (23.3)	83.28 (21.4)	91.64 (24.6)	91.58 (23.5)	91.24 (22.9)	85.45 (22.1)	79.25 (20.3)
**BMI in kg/m**^**2**^**, mean (SD)**	30.6 (7.3)	30.5 (7.2)	29.2 (6.7)	32.4 (8.0)	31.3 (7.2)	31.6 (7.0)	29.4 (6.8)	27.8 (6.6)
**BMI category, *N* (%)**								
Normal weight (< 25 kg/m^2^)	30,762 (22)	4103 (22)	1795 (28)	2733 (16)	8626 (17)	1465 (15)	5927 (27)	6113 (39)
Overweight (25.0– < 30 kg/m^2^)	44,809 (32)	6000 (32)	2173 (34)	4892 (28)	16,543 (32)	3096 (32)	7306 (33)	4799 (31)
Obesity (≥ 30.0 kg/m^2^)	65,689 (47)	8790 (47)	2407 (38)	9701 (56)	26,333 (51)	5134 (53)	8621 (39)	4703 (30)
**Hypertension diagnosis, *N* (%)**	88,380 (63)	15,052 (80)	2953 (46)	11,737 (68)	39,231 (76)	7149 (74)	10,471 (48)	1787 (11)
**Systolic BP ≥ 160 mmHg or diastolic BP ≥ 100 mmHg, *N* (%)**	26,984 (19)	5655 (30)	684 (11)	3194 (18)	12,538 (24)	1752 (18)	2590 (12)	571 (4)

Abbreviation: *Hctz*, hydrochlorothiazide

**Table 3:** Per-Protocol Effects: Absolute Weight Change, Relative Weight Change Compared to Lisinopril, and Relative Risk (RR) of Gaining ≥ 5% Baseline Weight Compared to Lisinopril Associated with Initiating and Adhering to Common Antihypertensive Medication over 6, 12, and 24 Months

**Absolute weight change (kg)**			
**Treatment**	**6 months**	**12 months**	**24 months**
	*β* (95% CI)	*β* (95% CI)	*β* (95% CI)
Lisinopril	** − 0.69 (− 0.92, − 0.47)**	** − 0.58 (− 1.05, − 0.30)**	** − 1.21 (− 2.13, − 0.46)**
Amlodipine	** − 0.43 (− 0.72, − 0.14)**	− 0.05 (− 0.64, 0.38)	0.82 (− 0.66, 2.00)
Atenolol	0.09 (− 0.72, 0.99)	0.29 (− 1.08, 1.73)	− 0.93 (− 2.30, 0.08)
Hctz	0.00 (− 0.29, 0.23)	** − 0.76 (− 1.49, − 0.13)**	− 0.57 (− 1.96, 0.76)
Losartan	− 0.15 (− 0.46, 0.19)	− 0.01 (− 0.68, 0.45)	** − 1.45 (− 3.03, − 0.21)**
Metoprolol	**0.70 (0.34, 0.98)**	**1.16 (0.53, 1.61)**	0.56 (− 1.15, 1.95)
Propranolol	0.34 (− 0.14, 0.87)	1.14 (− 0.49, 2.69)	1.14 (− 1.47, 3.03)
**Relative weight change (kg) compared to lisinopril**			
**Treatment**	**6 months**	**12 months**	**24 months**
	*β* (95% CI)	*β* (95% CI)	*β* (95% CI)
Lisinopril	0.0 (ref)	0.0 (ref)	0.0 (ref)
Amlodipine	0.25 (− 0.08, 0.62)	0.53 (− 0.15, 1.17)	**2.03 (0.50, 3.57)**
Atenolol	0.78 (− 0.06, 1.71)	0.87 (− 0.50, 2.42)	0.28 (− 1.24, 1.70)
Hctz	**0.68 (0.31, 1.04)**	− 0.18 (− 0.92, 0.70)	0.64 (− 1.00, 2.38)
Losartan	**0.54 (0.17, 0.93)**	0.57 (− 0.14, 1.23)	− 0.23 (− 1.99, 1.36)
Metoprolol	**1.38 (0.95, 1.76)**	**1.74 (1.03, 2.41)**	1.77 (− 0.07, 3.45)
Propranolol	**1.03 (0.46, 1.62)**	**1.72 (0.06, 3.35)**	2.35 (− 0.03, 4.43)
**Relative risk of gaining ≥ 5% baseline weight compared to lisinopril**			
**Treatment**	**6 months**	**12 months**	**24 months**
	RR (95% CI)	RR (95% CI)	RR (95% CI)
Lisinopril	1.0 (ref)	1.0 (ref)	1.0 (ref)
Amlodipine	1.18 (0.94, 1.51)	1.25 (0.86, 1.81)	1.26 (0.71, 2.10)
Atenolol	**1.68 (1.13, 2.67)**	1.00 (0.47, 1.91)	0.50 (0.15, 1.28)
Hctz	1.24 (0.97, 1.58)	1.03 (0.73, 1.48)	1.15 (0.64, 1.88)
Losartan	1.19 (0.91, 1.58)	0.95 (0.59, 1.49)	0.80 (0.40, 1.45)
Metoprolol	**1.67 (1.33, 2.19)**	**1.55 (1.18, 2.25)**	1.43 (0.87, 2.42)
Propranolol	**1.33 (1.04, 1.78)**	1.43 (0.98, 2.10)	1.63 (0.76, 3.21)

Abbreviation: *Hctz*, hydrochlorothiazide

Bold font indicates statistically significant weight changes (*p* < 0.05)

Stratification by baseline weight status (obesity vs. non-obesity) showed greater absolute weight change for patients without obesity across all medications (Table 4). Compared to lisinopril, patients with obesity had slightly greater weight change with amlodipine, metoprolol, and propranolol but slightly lower weight change with atenolol and hydrochlorothiazide. The differences in relative weight change were generally under 0.5 kg (Table 4).

**Table 4:** Stratified Per-Protocol Effects: Absolute Weight Change and Relative Weight Change Compared to Lisinopril Associated with Initiating and Adhering to Common Antihypertensive Medication over 6, 12, and 24 Months, Stratified by Baseline Obesity Status

**Absolute weight change (kg)**						
	**BMI < 30 kg/m**^**2**^			**BMI ≥ 30 kg/m**^**2**^		
**Treatment**	**6 months**	**12 months**	**24 months**	**6 months**	**12 months**	**24 months**
	*β* (95% CI)	*β* (95% CI)	*β* (95% CI)	*β* (95% CI)	*β* (95% CI)	*β* (95% CI)
Lisinopril	** − 0.13 (− 0.16, − 0.08)**	** − 0.03 (− 0.17, − 0.06)**	** − 0.65 (− 0.82, − 0.62)**	** − 1.30 (− 1.30, − 1.22)**	** − 1.20 (− 1.29, − 1.13)**	** − 1.83 (− 1.93, − 1.71)**
Amlodipine	**0.04 (0.00, 0.09)**	**0.43 (0.27, 0.38)**	**1.30 (0.87, 1.26)**	** − 0.96 (− 0.96, − 0.84)**	** − 0.57 (− 0.65, − 0.50)**	0.30 (− 0.04, 0.30)
Atenolol	**0.73 (0.60, 0.92)**	**0.91 (0.59, 0.95)**	** − 0.30 (− 0.55, − 0.31)**	** − 0.70 (− 0.80, − 0.45)**	** − 0.52 (− 0.78, − 0.20)**	** − 1.73 (− 1.98, − 1.59)**
Hctz	**0.64 (0.52, 0.70)**	** − 0.13 (− 0.23, − 0.13)**	0.05 (− 0.30, 0.05)	** − 0.65 (− 0.68, − 0.57)**	** − 1.42 (− 1.47, − 1.30)**	** − 1.24 (− 1.49, − 1.10)**
Losartan	**0.38 (0.32, 0.44)**	**0.52 (0.37, 0.50)**	** − 0.91 (− 1.16, − 0.76)**	** − 0.77 (− 0.80, − 0.63)**	** − 0.63 (− 0.75, − 0.57)**	** − 2.07 (− 2.29, − 1.78)**
Metoprolol	**1.03 (0.96, 1.08)**	**1.49 (1.35, 1.46)**	**0.89 (0.36, 0.91)**	**0.35 (0.30, 0.39)**	**0.81 (0.71, 0.82)**	0.22 (− 0.26, 0.25)
Propranolol	**0.76 (0.71, 0.84)**	**1.55 (1.30, 1.59)**	**1.57 (1.04, 1.69)**	− 0.13 (− 0.22, 0.01)	**0.67 (0.32, 0.74)**	**0.69 (0.18, 0.79)**
**Relative weight change (kg) compared to lisinopril**						
	**BMI < 30 kg/m**^**2**^			**BMI ≥ 30 kg/m**^**2**^		
**Treatment**	**6 months**	**12 months**	**24 months**	**6 months**	**12 months**	**24 months**
	*β* (95% CI)	*β* (95% CI)	*β* (95% CI)	*β* (95% CI)	*β* (95% CI)	*β* (95% CI)
Lisinopril	0.0 (ref)	0.0 (ref)	0.0 (ref)	0.0 (ref)	0.0 (ref)	0.0 (ref)
Amlodipine	**0.16 (0.11, 0.25)**	**0.46 (0.35, 0.51)**	**1.95 (1.54, 2.01)**	**0.34 (0.26, 0.42)**	**0.63 (0.50, 0.71)**	**2.13 (1.74, 2.22)**
Atenolol	**0.86 (0.68, 1.05)**	**0.94 (0.67, 1.01)**	**0.35 (0.07, 0.40)**	**0.61 (0.41, 0.84)**	**0.68 (0.39, 0.95)**	0.10 (− 0.18, 0.30)
Hctz	**0.76 (0.64, 0.83)**	** − 0.10 (− 0.14, − 0.02)**	**0.70 (0.40, 0.76)**	**0.65 (0.57, 0.69)**	** − 0.22 (− 0.31, − 0.04)**	**0.58 (0.35, 0.69)**
Losartan	**0.51 (0.41, 0.57)**	**0.55 (0.46, 0.59)**	** − 0.27 (− 0.51, − 0.05)**	**0.54 (0.45, 0.65)**	**0.58 (0.42, 0.67)**	− 0.24 (− 0.54, 0.10)
Metoprolol	**1.15 (1.08, 1.19)**	**1.51 (1.43, 1.60)**	**1.54 (1.10, 1.71)**	**1.66 (1.52, 1.65)**	**2.01 (1.88, 2.10)**	**2.05 (1.55, 2.13)**
Propranolol	**0.89 (0.85, 0.97)**	**1.58 (1.39, 1.70)**	**2.22 (1.79, 2.41)**	**1.18 (1.05, 1.28)**	**1.87 (1.49, 1.97)**	**2.52 (2.03, 2.68)**

Bold font indicates statistically significant weight changes (*p* < 0.05)

Additional stratified analyses by sex, race, and a proxy for menopausal status (women < 55 or ≥ 55 years) revealed some differences in weight change outcomes. When compared to patients on lisinopril as the reference medication, males had significantly higher weight change with atenolol, but females did not; female patients on losartan and propranolol had higher weight change at 6 months, but males did not (Table S5). Compared to patients on lisinopril, White individuals had significantly higher weight change with atenolol at 12 months and with propranolol at 6 months and 12 months, but non-White individuals did not (Table S6). Older females (≥ 55 years) had significantly greater weight change with atenolol and hydrochlorothiazide at 6 months compared to lisinopril, but younger females (< 55 years) did not; younger females had greater weight change with propranolol at 6 months compared to lisinopril, but not older females did not (Table S7).

The IO effects (Table S8) mirrored these findings from the PP analyses, but, for most comparisons, showed attenuated absolute weight change (see Tables 3 and S8 for details). IO effect estimates were also, as expected, generally more precise because their estimation did not require artificial censoring, and all patients were included in analyses. Analyses limited to patients with hypertension diagnoses or documented elevated blood pressure showed similar results (Table S9).

## DISCUSSION

In this target trial emulation study assessing the initiation of and adherence to common antihypertensive medications and population average weight change, we estimated that treating adults with lisinopril and amlodipine was associated with an average − 0.69 kg and − 0.43 kg weight loss after 6 months, respectively, while treatment with metoprolol was associated with a mean weight gain of 0.70 kg, consistent with our hypothesis. Compared to lisinopril, we estimated hydrochlorothiazide, losartan, metoprolol, and propranolol would be associated with 0.54–1.38 kg greater mean weight change at 6 months. Weight differences increased to 1.72 and 1.74 kg (propranolol and metoprolol) by 12 months and 2.03 kg (amlodipine) by 24 months. In IO analyses, absolute weight change estimates were attenuated as expected compared to PP analyses, as expected, because PP analyses considered scenarios with nonadherence to the initiated medication after baseline. We observed some modest differences in analyses stratified by subgroups (obesity, sex, race, age); results were unchanged when restricting to patients with hypertension or elevated blood pressure.

Our findings are consistent with previous research on weight change with use of lisinopril and beta-blockers. Lisinopril has led to weight loss in some RCTs.^12,33^ In one study of 126 adults with obesity and elevated diastolic blood pressure (*n* = 77 randomized to lisinopril), lisinopril treatment for 12 weeks resulted in a mean weight change of − 0.4 kg compared to baseline.^12^ A more recent trial of 51 female patients with hypertension and metabolic syndrome did not find any significant weight change after 12 weeks of lisinopril treatment.^11^ In contrast, beta-blockers have been linked to weight gain^10^, including among individuals with comorbid hypertension and diabetes.^34^ This weight change may result from metabolic alterations, including decreased basal metabolic rate and increased insulin resistance, as well as reductions in physical activity secondary to fatigue or shortness of breath.^10^ A meta-analysis of 8 prospective RCTs lasting at least 6 months found a mean weight gain of 1.2 kg in patients using beta-blockers (metoprolol, acebutolol, atenolol, propranolol) compared to control groups.^7^ Our analysis showed a similar pattern of weight gain for metoprolol and propranolol. Estimates for atenolol were imprecise, with large confidence intervals.

Prior evidence regarding weight change with amlodipine, hydrochlorothiazide, and losartan is less clear. One small RCT of 15 patients with hypertension and insulin resistance reported weight gain after 8 weeks of amlodipine treatment;^13^ other RCTs reported no weight change.^14,35,36^ Our analysis found a small weight loss at 6 months, but the weight loss did not persist at 12 and 24 months. Similar to studies of other diuretics, hydrochlorothiazide has been associated with modest weight loss; our study found no weight change.^12,37–39^ The evidence for losartan’s effect on weight is mixed, with some studies reporting weight loss.^40–43^ However, in two of these studies, patients receiving losartan also received diet and lifestyle interventions;^40–42^ patients in another study only included patients with cirrhosis.^43^ In a RCT with 81 patients with diabetic nephropathy, losartan was not associated with weight change.^44^ Our study found weight loss with losartan only at 24 months.

To our knowledge, this is the first study using a target trial approach to examine the association between initiation and adherence to common antihypertensive medications and weight change. This method analyzes observational data by explicitly considering causal effects and accounting for the dependence between covariates, exposures, and outcomes over time.^15,16,21^ Despite seemingly small observed weight differences, the effects could be meaningful at a population level due to the widespread use of these medications (63% of US adults take antihypertensives).^45^ Furthermore, we estimated significant differences in gaining 5% of baseline weight by 6 and 12 months for some beta-blockers, suggesting that a subset of patients had more substantial weight increases that could have important clinical implications. Strengths of this study include its large, diverse sample from 8 health systems, offering greater generalizability than previous studies.

Our study had several limitations. First, despite using more robust methods (i.e., “target trial emulation”) for the control of measured confounding and other threats to valid causal inference, our study is still an observational study; therefore, unmeasured/residual confounding (e.g., clinician preferences or unmeasured patient characteristics) may persist. Second, we only captured medication use and outcomes from the health systems we had access to, potentially missing prescriptions from other sources and biasing results towards the null. Misclassification of new users (80% accuracy) may dilute observed weight changes, as continuing users may have been more likely to have achieved stable weight or may have been less likely to experience fluctuations in weight upon initiation. Additionally, our study is limited by the fact that most patients did not have encounters with the health system exactly at 6, 12, or 24 months; specifically, only 15% to 25% had weight measurements recorded at those time points. To address this, we applied IPW, which adjusted for demographic factors, health behaviors, diagnoses, and medications predictive of having a weight measurement, as recommended for RCTs with incomplete outcome data.^46,47^ Third, low adherence after 2 years affected our ability to assess long-term effects and reduced precision at 24 months, with wider confidence intervals due to fewer patients in the analysis. However, both PP and IO analyses showed consistent results in magnitude and direction, with PP estimates further from the null, supporting the robustness of our findings. Fourth, inconsistent dosing information and missing data on prescription durations hindered our ability to evaluate dosage effects, and we could not confirm if patients actually filled their prescriptions. Fifth, while most medications are used to treat hypertension, some, like beta-blockers, have other uses. We conducted sensitivity analyses limiting the study population to patients with ICD codes for hypertension or elevated blood pressure measurements at baseline (systolic ≥ 140 mmHg or diastolic ≥ 90 mmHg) to address this; results were consistent with primary analyses. Sixth, weight change may be associated with nonadherence, and inaccuracies in EHR weight measurements and death records could have led to misclassification of nonadherence. Further, time-varying interim weight change itself may be a shared cause (i.e., confounder) of nonadherence and later weight change. To address this, our IP weights for nonadherence in our PP analysis included adjustment for baseline weight along with time-varying measures of last measured weight (kg) values. However, because weight is not consistently measured at all times in EHR data, this is a possible source of unmeasured confounding in our PP analyses. Last, our study focused exclusively on monotherapy, which may not reflect real-world prescribing patterns, as many patients with hypertension require combination therapy. Future research should examine the effects of polytherapy, particularly combinations of medications associated with weight gain, to better understand their cumulative impact on weight change and clinical outcomes.

## CONCLUSION

We estimated a small weight loss with lisinopril and amlodipine and modest weight gain with metoprolol 6 months after initiation and adherence. Hydrochlorothiazide, losartan, metoprolol, and propranolol were associated with greater weight gain compared to lisinopril.

## Supplementary Information

Below is the link to the electronic supplementary material.

Supplementary file1 (DOCX 94.1 KB)

## Data Availability

The data used in this article cannot be made public due to PCORnet regulations aimed at safeguarding the privacy of study participants. However, researchers can access PCORnet data for research purposes by following the formal policies and procedures set by PCORnet.

## Abbreviations

ACE: Angiotensin-converting enzyme
ARB: Angiotensin II receptor blocker
BMI: Body mass index
EHR: Electronic health record
ICD: International Classification of Diseases
IPW: Inverse probability weighting
IO: Initiation-only
NIDDK: National Institute of Diabetes and Digestive and Kidney Diseases
PCORnet: National Patient-Centered Clinical Research Network
PP: Per-protocol
RCT: Randomized controlled trial
RR: Relative risk
