# Supplementary material for: Emulation of a Target Trial of Antihypertensive Medications on Weight Change
Source: J Gen Intern Med. 2025 Sep 29;41(4):1097–106. doi: 10.1007/s11606-025-09787-x (PMC13009417; doi:10.1007/s11606-025-09787-x)
Supplement: Supplementary file 1 — Supplementary file1 (DOCX 94.1 KB) [file 11606_2025_9787_MOESM1_ESM.docx]

**Supplemental Online Materials**

**Title:** Emulation of a target trial comparing common antihypertensive medications on weight change

**Authors**: Pi-I Debby Lin, ScD, Sheryl L. Rifas-Shiman, MPH, Joshua Petimar, ScD, Han Yu, PhD, Matthew F. Daley, MD, David M. Janicke, PhD, ABPP, William J. Heerman, MD MPH, L. Charles Bailey, MD, PhD, Carlos Maeztu, MA, Kristina H. Lewis, MD, SM, MPH, Jessica G. Young, PhD, Jason P. Block, MD, MPH

**Contents:**

[**Figure S1.** Study flow chart 2](#_Toc198543013)

[**Table S1.** Comparison of Intent-to-Observe (IO) and Per-Protocol (PP) Analyses 4](#_Toc198543014)

[**Table S2.** Healthcare institutions included in the analysis. 5](#_Toc198543015)

[**Table S3.** Complete list of covariates included in analyses. 6](#_Toc198543016)

[**Table S4.** Number and proportion of patients who were adherent to each medication at 6, 12, and 24 months after initiation. 8](#_Toc198543017)

[**Table S5.** Stratified per-protocol effects: absolute weight change and relative weight change compared to lisinopril associated with initiating and adhering to common antihypertensive medication over 6, 12, and 24 months, stratified by sex 9](#_Toc198543018)

[**Table S6.** Stratified per-protocol effects: absolute weight change and relative weight change compared to lisinopril associated with initiating and adhering to common antihypertensive medication over 6, 12, and 24 months, stratified by race 10](#_Toc198543019)

[**Table S7** Stratified per-protocol effects: absolute weight change and relative weight change compared to lisinopril associated with initiating and adhering to common antihypertensive medication over 6, 12, and 24 months, stratified by proxy for menopause status (women <55 years or ≥55 years) 11](#_Toc198543020)

[**Table S9.** Sensitivity analysis on per-protocol effects: absolute weight change and relative weight change compared to lisinopril among patients initiating and adhering to common antihypertensive medication after 6, 12, and 24 months, limited to patients with a baseline hypertension diagnosis or elevated blood pressure (systolic ≥140 mmHg or diastolic ≥90 mmHg) within 6 months prior to medication initiation (n=110,721). 13](#_Toc198543021)

# **Figure S1.** Study flow chart

1. Sample size based on sequential exclusion.

n =142,734

2,355,075 study participants who were prescribed any antihypertensive medications.

Excluded 765,939 (did not initiate medication during study period)

n =1,589,136

Excluded 198,211 (not 20-80 years at initiation)

n =1,390,925

Excluded 299,636 (>1 antihypertensive medication at initiation)

n =1,091,289

Excluded 401,149 (did not initiate 1 of 7 specified medications)

n =690,140

Excluded 420,868 (no visit within 6 months prior to initiation)

n =269,272

Excluded 76,580 (no BMI within 3 months prior to initiation)

n =192,692

Excluded 16,289 (cancer diagnosis 1 year before to 1 month after initiation)

n 176,403

Excluded 2,750 (pregnant within 1 year prior to initiation)

n =173,653

Excluded 1,236 (bariatric surgery 3 year before to 1 month after initiation)

n =172,417

Excluded 5,639 (heart failure 1 year before to 1 months after initiation)

n =166,778

Excluded 1,187 (weight loss medication 1 months before to 1 month after initiation)

n =165,591

Excluded 12,775 (steroids 1month before to 1 month after initiation)

n =152,816

Excluded 1,932 (stimulant 1 month before to 1 month after initiation)

n =150,884

Excluded 8,147 (missing race or ethnicity)

n =142,737

Excluded 3 (missing sex)

Excluded 1,474 (no blood pressure within 6 months prior to initiation)

n =141,260

1. Total sample excluded by each criterion, non-sequential (N=2,355,075 who were prescribed any antihypertensive medications).

| **N** | **Criteria** |
| --- | --- |
| 323,226 | excluded if initiation not during study period |
| 291,557 | excluded if not 20-80y at initiation |
| 533,450 | excluded if >1 antihypertensive medication at initiation |
| 1,007,543 | excluded if did not initiate 1 of 7 specified medications |
| 1,477,515 | excluded in no visit within 6 months prior to initiation |
| 995,392 | excluded if no BMI within 3 months prior to initiation |
| 181,572 | excluded if cancer diagnosis 1 year before to 1 month after initiation |
| 37,888 | excluded if pregnant within 1 year prior to initiation |
| 8,822 | excluded if bariatric surgery 3 years before to 1 month after initiation |
| 168,509 | excluded if heart failure 1 year before to 1 month after initiation |
| 12,534 | excluded if weight loss medication 1 month before to 1 month after initiation |
| 228,450 | excluded if steroids 1 month before to 1 month after initiation |
| 35,600 | excluded if stimulant 1 month before to 1 month after initiation |
| 159,199 | excluded if race or ethnicity missing |
| 65 | excluded if sex missing |
| 442,715 | excluded 3 sites that did not have adequate data coverage during specific years |

# **Table S1.** Comparison of Intent-to-Observe (IO) and Per-Protocol (PP) Analyses

| **Aspect** | **Intent-to-Observe (IO) Analysis** | **Per-Protocol (PP) Analysis** |
| --- | --- | --- |
| **Goal/target of the analysis** | Structured to target the effect of treatment initiation only, regardless of adherence after baseline. This could be estimated unbiasedly in an ideal execution of the target trial by a simple comparison of mean weight change at the selected outcome time periods across treatment arms provided everyone initiated their treatment assignment at baseline only (regardless of adherence thereafter) and outcome measurement was complete. | Structured to target the effect of treatment under continuous adherence to the protocol over time. This could only be unbiasedly estimated by this simple comparison of means under full adherence to the treatment protocol over time and complete outcome measurement. |
| **Censoring** | No censoring when an individual’s data first becomes inconsistent with protocol adherence; all follow-up data is used regardless of adherence status. | An individual’s follow-up data is censored as soon as it becomes inconsistent with the treatment protocol (e.g., stop or switch medications). |
| **Inverse Probability Weighting (IPW)** | Adjusts for informative outcome measurement using baseline and time-varying covariates. | Adjusts for both informative outcome measurement and selection bias due to censoring for nonadherence. |
| **Strengths** | Reflects real-world scenarios where adherence may vary; useful for policy and decision-making. | Provides insights into the effects of treatment under optimal adherence, reducing bias from nonadherence. |
| **Limitations** | May capture effects of differential adherence across treatments, potentially biasing results. | May be less useful for policy and decision making. Stronger assumptions required for unbiased estimation of the target effect. |
| **Modeling Approach** | Uses IPW to adjust for missing outcome data and fits a repeated outcomes model with all follow-up. | Uses IPW to adjust for missing outcome data and censoring, then fits a repeated outcomes model to censored data. |

# **Table S2.** Healthcare institutions included in the analysis.

| Site | Years included^1^ | Sample size |
| --- | --- | --- |
| Kaiser Permanente Colorado | 2010-2019 | 33,049 |
| Duke University Medical Center | 2014-2019 | 38,275 |
| Vanderbilt University Medical Center | 2010-2019 | 26,644 |
| University of Florida | 2015-2019 | 18,180 |
| AdventHealth | 2014-2019 | 7,616 |
| University of Miami | 2012-2019 | 4,926 |
| Tallahassee Memorial HealthCare | 2012-2019 | 7,104 |
| Orlando Health System | 2012-2019 | 5,466 |
| ^1^Dates of medication availability |  |  |

# **Table S3.** Complete list of covariates included in analyses.

| **Covariate** | **Adjusted for baseline?** | **Adjusted for over follow-up?** | **Terms^1^** |
| --- | --- | --- | --- |
| Age at baseline | Yes | No | Continuous spline |
| Sex | Yes | No | Male, Female |
| Race | Yes | No | White, Black, Asian-American/Pacific Islander, Other (including other races, >1 races) |
| Hispanic ethnicity | Yes | No | Yes, No |
| Site | Yes | No | Indicator for each site |
| Year of initiation | Yes | No | Indicator for each year |
| SBP ≥160 or DBP ≥100 | Yes | No | Yes, No within 6 months before initiation |
|  |  |  |  |
|  |  |  |  |
| BMI trajectory | Yes | Yes | Gaining weight, losing weight, stable weight |
| BMI | Yes | Yes | Continuous spline |
|  |  |  |  |
|  |  |  |  |
| Medicaid status | Yes | Yes | Yes, No |
| Smoking status | Yes | Yes | Yes, No |
| Weight loss medication prescription |  | Yes | Rx in current month, no rx in current month |
| Stimulants prescription |  | Yes | Rx in current month, no rx in current month |
| Steroid prescription |  | Yes | Rx in current month, no rx in current month |
| Statin prescription | No | Yes | Rx in current month, no rx in current month |
| Hypertension diagnosis | Yes | Yes | Dx <12 months ago + dx ≥12 months ago + interaction |
| Hyperthyroidism diagnosis | Yes | Yes | Dx <12 months ago + dx ≥12 months ago + interaction |
| Hypothyroidism diagnosis | Yes | Yes | Dx <12 months ago + dx ≥12 months ago + interaction |
|  |  |  |  |
| Type 2 diabetes diagnosis | Yes | Yes | Dx <12 months ago + dx ≥12 months ago + interaction |
| Growth condition diagnosis | Yes | Yes | Dx <12 months ago + dx ≥12 months ago + interaction |
| Abnormal glucose diagnosis | Yes | Yes | Dx <12 months ago + dx ≥12 months ago + interaction |
| Polycystic ovary syndrome diagnosis | Yes | Yes | Dx <12 months ago + dx ≥12 months ago + interaction |
| Acute myocardial infarction | Yes | Yes | Dx <12 months ago + dx ≥12 months ago + interaction |
| Ischemic stroke | Yes | Yes | Dx <12 months ago + dx ≥12 months ago + interaction |
| Hemorrhagic stroke | Yes | Yes | Dx <12 months ago + dx ≥12 months ago + interaction |
| Coronary artery disease | Yes | Yes | Dx <12 months ago + dx ≥12 months ago + interaction |
| Heart failure diagnosis^2^ | Yes | Yes | Dx <12 months ago + dx ≥12 months ago + interaction |
| Chronic kidney disease diagnosis | Yes | Yes | Dx <12 months ago + dx ≥12 months ago + interaction |
| Carlson comorbidity index >1 | Yes | Yes | Dx <12 months ago + dx ≥12 months ago + interaction |
| Alcohol abuse diagnosis | Yes | Yes | Dx <12 months ago + dx ≥12 months ago + interaction |
| Liver cirrhosis diagnosis | Yes | Yes | Dx <12 months ago + dx ≥12 months ago + interaction |
| Drug abuse diagnosis | Yes | Yes | Dx <12 months ago + dx ≥12 months ago + interaction |
| Cancer diagnosis | No | Yes | Dx <12 months ago + dx ≥12 months ago + interaction |
| Bariatric surgery | No | Yes | Dx <12 months ago + dx ≥12 months ago + interaction |
| Pregnancy | No | Yes | Dx <12 months ago + dx ≥12 months ago + interaction |
| Antiseizure medication prescription^3^ | Yes | Yes | Rx <15 months ago |
| Diabetes medication prescription ^3^ | Yes | Yes | Rx <15 months ago |
| Antipsychotic medication prescription^3^ | Yes | Yes | Rx <15 months ago |
| Antidepressant medication prescription^3^ | Yes | Yes | Rx <15 months ago |
| Other antihypertensive prescription^4^ | No | Yes | Rx in current month, no rx in current month |
| Number of encounters | Yes | Yes | Categorical number of health system encounters |
| ^1^Diagnosis variables included terms for dx <12 months ago to adjust for recent history, dx ≥12 months ago to adjust for older history, and an interaction term to adjust for having both a recent and an older history with the condition. | | | |
| ^2^For baseline adjustment, the only term included for heart failure dx was ≥12 months ago because a person with a heart failure dx <12 months before initiation was ineligible for inclusion | | | |
| ^3^15 months was used because we assumed, conservatively, that each patient could be given a prescription for up to 12 months. We added an additional 3 months as a grace period (i.e., allowing each patient up to 3 months to refill the prescription). | | | |
| ^4^Using the same rules for calculating the length of time of each prescription as for the main treatment strategy. | | | |

# **Table S4.** Number and proportion of patients who were adherent to each medication at 6, 12, and 24 months after initiation.

|  | **Amlodipine** | | |
| --- | --- | --- | --- |
|  | 6m | 12m | 24m |
| N | 6638 | 4644 | 1027 |
| % | 35% | 25% | 5% |
|  | **Atenolol** | | |
|  | 6m | 12m | 24m |
| N | 1277 | 857 | 207 |
| % | 20% | 13% | 3% |
|  | **Hctz** | | |
|  | 6m | 12m | 24m |
| N | 6456 | 4595 | 966 |
| % | 37% | 27% | 6% |
|  | **Lisinopril** | | |
|  | 6m | 12m | 24m |
| N | 15509 | 10389 | 2337 |
| % | 30% | 20% | 5% |
|  | **Losartan** | | |
|  | 6m | 12m | 24m |
| N | 4054 | 2907 | 631 |
| % | 42% | 30% | 7% |
|  | **Metoprolol** | | |
|  | 6m | 12m | 24m |
| N | 5822 | 4175 | 865 |
| % | 27% | 19% | 4% |
|  | **Propranolol** | | |
|  | 6m | 12m | 24m |
| N | 5082 | 2947 | 681 |
| % | 33% | 19% | 4% |

Abbreviation: Hctz: hydrochlorothiazide.

# **Table S5.** Stratified per-protocol effects: absolute weight change and relative weight change compared to lisinopril associated with initiating and adhering to common antihypertensive medication over 6, 12, and 24 months, stratified by sex

| **Absolute weight change (kg)** | | | | | | |
| --- | --- | --- | --- | --- | --- | --- |
|  | **Male** | | | **Female** | | |
| **Treatment** | **6 months** | **12 months** | **24 months** | **6 months** | **12 months** | **24 months** |
|  | β (95% CI) | β (95% CI) | β (95% CI) | β (95% CI) | β (95% CI) | β (95% CI) |
| Lisinopril | **-0.66 (-0.96,-0.39)** | **-0.72 (-1.23,-0.28)** | **-1.06 (-2.04,-0.12)** | **-0.67 (-1.03,-0.37)** | **-0.53 (-1.25,-0.06)** | -1.13 (-2.67, 0.18) |
| Amlodipine | **-0.42 (-0.79,-0.04)** | **-0.56 (-1.34, 0.00)** | -0.07 (-1.38, 1.30) | -0.38 (-0.82, 0.01) | 0.30 (-0.48, 1.00) | 0.95 (-0.78, 2.44) |
| Atenolol | **2.28 (0.98, 3.42)** | -0.21 (-1.80, 1.05) | **-1.96 (-5.06,-0.18)** | -0.77 (-1.59, 0.31) | 0.52 (-1.48, 2.40) | -0.69 (-2.11, 0.77) |
| Hctz | 0.11 (-0.38, 0.58) | 0.00 (-0.84, 0.72) | -0.53 (-2.60, 2.05) | -0.02 (-0.37, 0.27) | **-1.16 (-2.18,-0.32)** | -0.72 (-2.52, 1.17) |
| Losartan | -0.33 (-0.72, 0.09) | -0.15 (-0.99, 0.64) | -1.25 (-3.59, 0.53) | 0.04 (-0.43, 0.46) | 0.11 (-0.69, 0.70) | **-1.23 (-2.60,-0.08)** |
| Metoprolol | **0.63 (0.18, 1.10)** | **1.28 (0.47, 2.06)** | 1.87 (-1.04, 4.35) | **0.67 (0.26, 1.02)** | **0.95 (0.22, 1.51)** | -0.32 (-1.87, 0.87) |
| Propranolol | -0.01 (-0.71, 0.84) | 1.01 (-0.37, 2.42) | 1.62 (-0.57, 3.26) | 0.42 (-0.13, 1.03) | 1.12 (-0.66, 2.86) | 0.82 (-2.04, 3.20) |
| **Relative weight change (kg) compared to lisinopril** | | | | | | |
|  | **Male** | | | **Female** | | |
| **Treatment** | **6 months** | **12 months** | **24 months** | **6 months** | **12 months** | **24 months** |
|  | β (95% CI) | β (95% CI) | β (95% CI) | β (95% CI) | β (95% CI) | β (95% CI) |
| Lisinopril | 0.0 (ref) | 0.0 (ref) | 0.0 (ref) | 0.0 (ref) | 0.0 (ref) | 0.0 (ref) |
| Amlodipine | 0.24 (-0.23, 0.70) | 0.16 (-0.70, 0.93) | 0.99 (-0.65, 2.69) | 0.28 (-0.23, 0.81) | 0.84 (-0.10, 1.86) | 2.08 (-0.20, 4.03) |
| Atenolol | **2.94 (1.56, 4.09)** | 0.51 (-1.14, 1.95) | -0.9 (-4.30, 1.17) | -0.11 (-0.98, 1.05) | 1.06 (-0.94, 3.11) | 0.44 (-1.59, 2.57) |
| Hctz | **0.77 (0.19, 1.31)** | 0.72 (-0.23, 1.61) | 0.54 (-1.74, 3.26) | **0.65 (0.19, 1.12)** | -0.62 (-1.77, 0.54) | 0.41 (-1.91, 2.86) |
| Losartan | 0.34 (-0.09, 0.84) | 0.56 (-0.40, 1.50) | -0.19 (-2.73, 1.89) | **0.70 (0.18, 1.22)** | 0.64 (-0.31, 1.56) | -0.10 (-2.21, 1.81) |
| Metoprolol | **1.30 (0.75, 1.84)** | **2.00 (1.02, 2.98)** | 2.93 (-0.27, 5.67) | **1.33 (0.85, 1.88)** | **1.48 (0.59, 2.45)** | 0.81 (-1.39, 2.67) |
| Propranolol | 0.66 (-0.15, 1.58) | **1.72 (0.24, 3.32)** | **2.68 (0.23, 4.57)** | **1.09 (0.46, 1.84)** | 1.66 (-0.16, 3.58) | 1.95 (-1.19, 4.76) |

Note: bold font indicates statistically significant weight changes (p<0.05).

# **Table S6.** Stratified per-protocol effects: absolute weight change and relative weight change compared to lisinopril associated with initiating and adhering to common antihypertensive medication over 6, 12, and 24 months, stratified by race

| **Absolute weight change (kg)** | | | | | | |
| --- | --- | --- | --- | --- | --- | --- |
|  | **Non-White** | | | **White** | | |
| **Treatment** | **6 months** | **12 months** | **24 months** | **6 months** | **12 months** | **24 months** |
|  | β (95% CI) | β (95% CI) | β (95% CI) | β (95% CI) | β (95% CI) | β (95% CI) |
| Lisinopril | **-0.78 (-1.11,-0.29)** | **-0.92 (-1.69,-0.23)** | **-1.71 (-3.44,-0.14)** | **-0.67 (-0.97,-0.42)** | **-0.49 (-1.02,-0.19)** | **-0.93 (-2.02,-0.11)** |
| Amlodipine | **-0.52 (-0.91, 0.00)** | -0.29 (-0.80, 0.29) | 1.2 (-1.10, 3.32) | **-0.39 (-0.72,-0.07)** | 0.05 (-0.79, 0.84) | 0.5 (-0.87, 1.66) |
| Atenolol | -0.46 (-1.43, 1.17) | -3.21 (-7.01, 0.19) | -3.35 (-6.82, 0.33) | 0.31 (-0.68, 1.28) | 0.85 (-0.42, 2.02) | -0.2 (-1.56, 0.72) |
| Hctz | 0.28 (-0.24, 0.76) | -0.43 (-1.33, 0.68) | 1.68 (-1.67, 4.48) | -0.19 (-0.57, 0.09) | **-0.99 (-2.04,-0.27)** | **-1.35 (-2.80,-0.01)** |
| Losartan | -0.13 (-0.55, 0.39) | -0.33 (-1.06, 0.44) | -1.00 (-3.48, 0.63) | -0.18 (-0.61, 0.22) | 0.09 (-0.71, 0.62) | -1.71 (-4.13, 0.05) |
| Metoprolol | **1.04 (0.39, 1.92)** | 0.53 (-0.61, 1.89) | -0.45 (-1.96, 1.19) | **0.55 (0.18, 0.82)** | **1.30 (0.57, 1.74)** | 0.51 (-1.28, 1.93) |
| Propranolol | -0.33 (-0.75, 0.62) | 0.32 (-0.78, 1.90) | **7.44 (1.83,11.88)** | 0.46 (-0.21, 1.06) | 1.43 (-0.60, 3.05) | 0.51 (-1.85, 2.38) |
| **Relative weight change (kg) compared to lisinopril** | | | | | | |
|  | **Non-White** | | | **White** | | |
| **Treatment** | **6 months** | **12 months** | **24 months** | **6 months** | **12 months** | **24 months** |
|  | β (95% CI) | β (95% CI) | β (95% CI) | β (95% CI) | β (95% CI) | β (95% CI) |
| Lisinopril | 0.0 (ref) | 0.0 (ref) | 0.0 (ref) | 0.0 (ref) | 0.0 (ref) | 0.0 (ref) |
| Amlodipine | 0.26 (-0.37, 0.90) | 0.64 (-0.26, 1.65) | 2.90 (-0.05, 5.50) | 0.28 (-0.12, 0.73) | 0.53 (-0.35, 1.51) | 1.43 (-0.22, 2.99) |
| Atenolol | 0.32 (-0.84, 1.93) | -2.29 (-6.10, 1.31) | -1.65 (-5.27, 2.26) | 0.97 (-0.08, 1.98) | **1.34 (0.09, 2.67)** | 0.73 (-0.82, 2.16) |
| Hctz | **1.06 (0.28, 1.70)** | 0.49 (-0.65, 1.92) | 3.38 (-0.49, 6.57) | **0.48 (0.04, 0.91)** | -0.50 (-1.64, 0.44) | -0.41 (-1.96, 1.38) |
| Losartan | **0.65 (0.02, 1.25)** | 0.59 (-0.45, 1.66) | 0.71 (-2.18, 3.13) | **0.49 (0.03, 0.99)** | 0.58 (-0.26, 1.34) | -0.78 (-3.31, 1.43) |
| Metoprolol | **1.82 (0.92, 2.79)** | **1.45 (0.02, 3.09)** | 1.25 (-1.07, 3.60) | **1.22 (0.78, 1.64)** | **1.78 (1.03, 2.48)** | 1.45 (-0.44, 3.22) |
| Propranolol | 0.45 (-0.20, 1.44) | 1.24 (-0.10, 3.10) | **9.14 (3.28,14.04)** | **1.12 (0.38, 1.87)** | **1.92 (0.00, 3.78)** | 1.44 (-0.91, 3.77) |

Note: bold font indicates statistically significant weight changes (p<0.05).

# **Table S7** Stratified per-protocol effects: absolute weight change and relative weight change compared to lisinopril associated with initiating and adhering to common antihypertensive medication over 6, 12, and 24 months, stratified by proxy for menopause status (women <55 years or ≥55 years)

| **Absolute weight change (kg)** | | | | | | |
| --- | --- | --- | --- | --- | --- | --- |
|  | **<55y** | | | **≥55y** | | |
| **Treatment** | **6 months** | **12 months** | **24 months** | **6 months** | **12 months** | **24 months** |
|  | β (95% CI) | β (95% CI) | β (95% CI) | β (95% CI) | β (95% CI) | β (95% CI) |
| Lisinopril | -0.48 (-1.07, 0.15) | -0.27 (-1.42, 0.52) | -1.91 (-4.69, 0.35) | **-0.78 (-1.12,-0.59)** | **-0.71 (-1.40,-0.20)** | -0.18 (-1.52, 1.07) |
| Amlodipine | -0.22 (-0.71, 0.34) | 0.73 (-0.44, 1.73) | 2.65 (-0.26, 5.31) | -0.42 (-1.05, 0.05) | -0.21 (-0.89, 0.35) | 0.07 (-1.29, 1.37) |
| Atenolol | -1.54 (-2.58, 0.22) | -0.08 (-4.91, 4.00) | -0.39 (-2.38, 1.97) | 0.08 (-0.74, 0.81) | 0.60 (-0.70, 1.40) | -0.42 (-2.52, 1.00) |
| Hctz | 0.08 (-0.45, 0.64) | **-2.42 (-3.98,-0.93)** | -0.62 (-4.24, 2.88) | -0.03 (-0.46, 0.24) | 0.09 (-0.67, 0.58) | -0.62 (-1.57, 0.16) |
| Losartan | 0.33 (-0.42, 1.14) | 0.65 (-0.37, 1.55) | -0.24 (-5.62, 2.99) | -0.12 (-0.64, 0.22) | -0.28 (-1.38, 0.49) | **-1.63 (-3.05,-0.36)** |
| Metoprolol | **0.65 (0.18, 1.30)** | 0.74 (-0.17, 1.70) | -0.9 (-4.66, 1.52) | **0.62 (0.01, 1.05)** | **1.02 (0.17, 1.75)** | 0.16 (-1.33, 1.52) |
| Propranolol | **0.61 (0.05, 1.27)** | 1.00 (-0.70, 2.80) | 0.08 (-5.26, 3.96) | -0.34 (-1.21, 0.56) | 1.69 (-1.87, 6.01) | 2.07 (-0.34, 3.86) |
| **Relative weight change (kg) compared to lisinopril** | | | | | | |
|  | **<55y** | | | **≥55y** | | |
| **Treatment** | **6 months** | **12 months** | **24 months** | **6 months** | **12 months** | **24 months** |
|  | β (95% CI) | β (95% CI) | β (95% CI) | β (95% CI) | β (95% CI) | β (95% CI) |
| Lisinopril | 0.0 (ref) | 0.0 (ref) | 0.0 (ref) | 0.0 (ref) | 0.0 (ref) | 0.0 (ref) |
| Amlodipine | 0.26 (-0.50, 1.07) | 1.01 (-0.33, 2.60) | **4.56 (0.64, 8.13)** | 0.36 (-0.24, 1.01) | 0.50 (-0.34, 1.39) | 0.25 (-1.67, 2.17) |
| Atenolol | -1.06 (-2.32, 0.92) | 0.19 (-4.49, 4.57) | 1.51 (-1.28, 5.16) | **0.86 (0.05, 1.69)** | 1.31 (-0.02, 2.39) | -0.24 (-2.48, 1.72) |
| Hctz | 0.56 (-0.18, 1.44) | **-2.14 (-4.01, 0.00)** | 1.29 (-2.97, 5.67) | **0.76 (0.27, 1.21)** | 0.80 (-0.14, 1.71) | -0.44 (-2.03, 1.14) |
| Losartan | 0.80 (-0.11, 1.79) | 0.92 (-0.19, 2.32) | 1.67 (-4.06, 6.10) | **0.66 (0.16, 1.15)** | 0.44 (-0.80, 1.47) | -1.44 (-3.51, 0.44) |
| Metoprolol | **1.13 (0.28, 2.09)** | 1.02 (-0.15, 2.59) | 1.01 (-3.38, 4.69) | **1.40 (0.77, 1.99)** | **1.74 (0.70, 2.82)** | 0.34 (-1.57, 2.26) |
| Propranolol | **1.09 (0.19, 2.05)** | 1.28 (-0.62, 3.47) | 1.99 (-3.82, 6.71) | 0.45 (-0.37, 1.52) | 2.4 (-1.24, 6.82) | 2.25 (-0.61, 4.58) |

Note: bold font indicates statistically significant weight changes (p<0.05).

**Table S8.** Initiation-only effects: absolute weight change and relative weight change compared to lisinopril among patients initiating common antihypertensive medications after 6, 12, and 24 months.

| **Absolute weight change (kg)** | | | |
| --- | --- | --- | --- |
| **Treatment** | **6 months** | **12 months** | **24 months** |
|  | β (95% CI) | β (95% CI) | β (95% CI) |
| Lisinopril | **-0.47 (-0.53,-0.41)** | **-0.38 (-0.47,-0.31)** | **-0.37 (-0.50,-0.27)** |
| Amlodipine | **-0.22 (-0.32,-0.13)** | **-0.15 (-0.29,-0.04)** | -0.11 (-0.31, 0.07) |
| Atenolol | **-0.23 (-0.40,-0.07)** | **0.23 (0.01, 0.44)** | **0.38 (0.09, 0.64)** |
| Hctz | **-0.33 (-0.43,-0.24)** | **-0.34 (-0.47,-0.21)** | **-0.30 (-0.51,-0.13)** |
| Losartan | 0.04 (-0.08, 0.16) | 0.07 (-0.09, 0.22) | **-0.34 (-0.59,-0.10)** |
| Metoprolol | 0.04 (-0.06, 0.12) | **0.23 (0.10, 0.36)** | **0.25 (0.07, 0.42)** |
| Propranolol | **-0.25 (-0.38,-0.15)** | 0.15 (-0.01, 0.28) | **0.62 (0.39, 0.81)** |
| **Relative weight change (kg) compared to lisinopril** | | | |
| **Treatment** | **6 months** | **12 months** | **24 months** |
|  | β (95% CI) | β (95% CI) | β (95% CI) |
| Lisinopril | 0.0 (ref) | 0.0 (ref) | 0.0 (ref) |
| Amlodipine | **0.25 (0.14, 0.36)** | **0.23 (0.07, 0.37)** | **0.27 (0.05, 0.49)** |
| Atenolol | **0.24 (0.07, 0.41)** | **0.62 (0.38, 0.82)** | **0.75 (0.46, 1.05)** |
| Hctz | **0.14 (0.03, 0.25)** | 0.05 (-0.10, 0.20) | 0.08 (-0.15, 0.28) |
| Losartan | **0.51 (0.38, 0.64)** | **0.45 (0.27, 0.62)** | 0.03 (-0.23, 0.29) |
| Metoprolol | **0.51 (0.39, 0.61)** | **0.62 (0.46, 0.76)** | **0.63 (0.42, 0.85)** |
| Propranolol | **0.22 (0.07, 0.35)** | **0.53 (0.36, 0.68)** | **1.00 (0.75, 1.23)** |

Abbreviation: Hctz: hydrochlorothiazide.

Note: bold font indicates statistically significant weight changes (p<0.05).

# **Table S9.** Sensitivity analysis on per-protocol effects: absolute weight change and relative weight change compared to lisinopril among patients initiating and adhering to common antihypertensive medication after 6, 12, and 24 months, limited to patients with a baseline hypertension diagnosis or elevated blood pressure (systolic ≥140 mmHg or diastolic ≥90 mmHg) within 6 months prior to medication initiation (n=110,721).

| **Absolute weight change (kg)** | | | |
| --- | --- | --- | --- |
| **Treatment** | **6 months** | **12 months** | **24 months** |
|  | β (95% CI) | β (95% CI) | β (95% CI) |
| Lisinopril | **-0.71 (-0.95,-0.50)** | **-0.67 (-1.13,-0.31)** | **-1.13 (-2.04,-0.29)** |
| Amlodipine | **-0.52 (-0.81,-0.21)** | -0.24 (-0.75, 0.24) | 0.81 (-0.66, 1.98) |
| Atenolol | 0.42 (-0.59, 1.43) | -0.25 (-1.33, 0.67) | -0.83 (-2.95, 0.50) |
| Hctz | -0.06 (-0.36, 0.20) | -0.62 (-1.42, 0.02) | -0.59 (-2.29, 0.99) |
| Losartan | -0.21 (-0.53, 0.09) | -0.23 (-0.79, 0.20) | **-1.53 (-3.06,-0.11)** |
| Metoprolol | **0.65 (0.25, 1.00)** | **1.04 (0.34, 1.60)** | 0.83 (-1.02, 2.54) |
| Propranolol | 0.11 (-0.60, 0.76) | 0.15 (-1.47, 1.73) | 1.19 (-1.69, 4.52) |
| **Relative weight change (kg) compared to lisinopril** | | | |
| **Treatment** | **6 months** | **12 months** | **24 months** |
|  | β (95% CI) | β (95% CI) | β (95% CI) |
| Lisinopril | 0.0 (ref) | 0.0 (ref) | 0.0 (ref) |
| Amlodipine | 0.19 (-0.15, 0.59) | 0.44 (-0.24, 1.10) | **1.94 (0.28, 3.41)** |
| Atenolol | **1.13 (0.14, 2.18)** | 0.42 (-0.70, 1.42) | 0.3 (-2.00, 2.05) |
| Hctz | **0.65 (0.29, 1.02)** | 0.05 (-0.85, 0.83) | 0.54 (-1.32, 2.47) |
| Losartan | **0.51 (0.11, 0.88)** | 0.44 (-0.16, 1.07) | -0.4 (-2.03, 1.41) |
| Metoprolol | **1.36 (0.93, 1.77)** | **1.71 (0.94, 2.49)** | 1.96 (-0.12, 3.89) |
| Propranolol | **0.82 (0.09, 1.51)** | 0.82 (-0.80, 2.51) | 2.32 (-0.63, 5.79) |

Abbreviation: Hctz: hydrochlorothiazide.

Note: bold font indicates statistically significant weight changes (p<0.05).
